# USP38 Couples Histone Ubiquitination and Methylation via KDM5B to Resolve Inflammation

**DOI:** 10.1002/advs.202101964

**Published:** 2021-06-24

**Authors:** Zhiyao Zhao, Zexiong Su, Puping Liang, Di Liu, Shuai Yang, Yaoxing Wu, Ling Ma, Junyan Feng, Xiya Zhang, Chenglei Wu, Junjiu Huang, Jun Cui

*Adv. Sci*. **2020**, *7*, 2002680

DOI: 10.1002/advs.202002680

**Figure S3:**
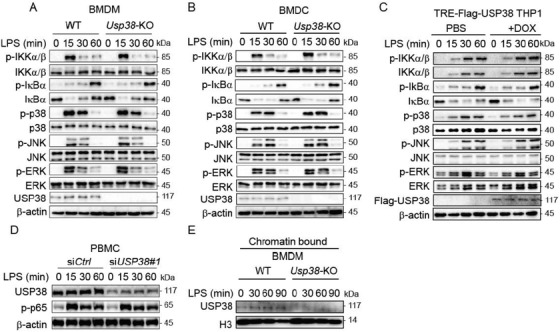
USP38 is dispensable for the TLR‐stimulated signaling cascade. (A‐B) Immunoblot analysis of phosphorylated (p‐) and total proteins in whole‐cell lysates of WT and Usp38‐KO BMDMs (A) and BMDCs (B) that were stimulated with LPS for the indicated time points. (C) Immunoblot analysis of phosphorylated (p‐) and total protein in whole‐cell lysates of USP38‐inducible THP1 cells with or without DOX that were stimulated with LPS for the indicated time points. (D) Immunoblot analysis of phosphorylated p65 in PBMCs silenced with control siRNA (siCtrl) or USP38‐specific siRNA (siUSP38) under LPS treatment for the indicated time points. (E) Immunoblot analysis of USP38 in the chromatin‐bound and unbound components of WT and Usp38‐KO BMDMs under LPS treatment for the indicated time points. Data are representative of 3 independent biological experiments.

In the originally published article, the same figure for Figure S3 A and B were shown (Supporting Information). Please find the correct Figure S3 here:

The authors apologize for any inconvenience this may have caused.

